# Methicillin resistance in *Staphylococcus pseudintermedius* encoded within novel staphylococcal cassette chromosome *mec* (SCC*mec*) variants

**DOI:** 10.1093/jac/dkae096

**Published:** 2024-04-02

**Authors:** A C MacFadyen, G K Paterson

**Affiliations:** Royal (Dick) School of Veterinary Studies and the Roslin Institute, University of Edinburgh, Easter Bush, Midlothian EH25 9RG, UK; Royal (Dick) School of Veterinary Studies and the Roslin Institute, University of Edinburgh, Easter Bush, Midlothian EH25 9RG, UK

## Abstract

**Background:**

*Staphylococcus pseudintermedius* is a common opportunistic pathogen of companion dogs and an occasional human pathogen. Treatment is hampered by antimicrobial resistance including methicillin resistance encoded by *mecA* within the mobile genetic element SCC*mec*.

**Objectives:**

SCC*mec* elements are diverse, especially in non-*Staphyloccocus aureus* staphylococci, and novel variants are likely to be present in *S*. *pseudintermedius*. The aim was to characterize the SCC*mec* elements found in four canine clinical isolates of *S*. *pseudintermedius*.

**Material and methods:**

Isolates were whole-genome sequenced and SCC*mec* elements were assembled, annotated and compared to known SCC*mec* types.

**Results and discussion:**

Two novel SSC*mec* are present in these isolates. SCC*mec*_7017-61515_ is characterized by a novel combination of a Class A *mec* gene complex and a type 5 *ccr* previously only described in composite SCC*mec* elements. The other three isolates share a novel composite SCC*mec* with features of SCC*mec* types IV and VI.

**Conclusions:**

*S*. *pseudintermedius* is a reservoir of novel SSC*mec* elements that has implications for understanding antimicrobial resistant in veterinary and human medicine.

## Introduction

*Staphylococcus pseudintermedius* a coagulase-positive staphylococci, previously confused with *Staphylococcus intermedius*, was first recognized as a separate species in 2005^1^ following the re-classification of *S*. *intermedius* into two species; *S*. *intermedius* and *S*. *pseudintermedius*. Together with *Staphylcococus delphini*^2^ and *Staphylococcus cornubiensis*^3^—both separately described—they form the *S*. *intermedius* group. A further proposed species, ‘*Staphylococcus ursi*’ (not validly published at the time writing) has been suggested as additional member of this group.^4^*S*. *pseudintermedius* is a common commensal of healthy companion dogs residing on the cutaneous and mucosal membranes, with the mouth, nose, groin and perineum–rectum the most commonly colonized regions.^5^ Although a part of the normal canine microbiota, *S*. *pseudintermedius* is a frequent opportunistic pathogen of dogs causing a range of infections, most commonly pyoderma, otitis externa, wound and surgical site infections, and urinary tract infections.^5–9^ Such is the prevalence of *S*. *pseudintermedius* canine pyoderma that it is a leading cause of antimicrobial prescriptions in small animal practice.^10^ Allied to this antimicrobial use is the problem of antimicrobial resistance in *S*. *pseudintermedius*, including methicillin resistance and multidrug resistance.^11–13^

Methicillin resistance in *S*. *pseudintermedius*, as in other staphylococci,^14^ is conferred by *mecA*,^15^ encoding an alternative penicillin-binding protein PBP2a.^16^*mecA* being carried by a variable mobile genetic element known as staphylococcal cassette chromosome *mec* (SCC*mec*).^17^ SCC*mec* diversity is best studied in *S*. *aureus* where 15 types, I–XV, are currently recognized with nomenclature being governed by the International Working Group on the Classification of Staphylococcal Cassette Chromosome Elements (IWG-SCC) (https://www.sccmec.org/ accessed on 1 May 2023) with several subtypes also being recognized.^18–22^ Despite the diversity among SCC*mec* elements, a number of shared features are used to define them; a *mec* gene complex, site-specific recombinases designated as cassette chromosome recombinases [*ccr* gene(s)], characteristic direct repeats and inverted repeats at both ends and integration into *orfX* encoding the ribosomal methyltransferase RlmH.^19–21^ SCC*mec* include joining (J) regions, defined by the areas between the *orfX*, *ccr* and *mec* genes. Often the components within the J regions are non-essential, however, they can contain determinants of antimicrobial resistance, thereby providing the SCC*mec* the ability to encode for additional resistance mechanisms beyond methicillin. SCC*mec* elements are found widely among the *Staphylococcus* genus as well as in the related Gram-stain positive genera *Mammaliicoccus* and *Macrococcus*.^23^ While some SCC*mec* elements found in *S*. *aureus* are also found in other species, the diversity of SCC*mec* elements is even greater among non-*S*. *aureus* organisms such that the ‘IWG-SCC has decided not to annotate new SCC*mec* subtypes in other species than *S*. *aureus*, due to the high complexity of the elements found in isolates other than *S*. *aureus*’.^21,24^ Instead an alternative nomenclature is ‘SCC*mec*[NAME OF THE STRAIN]’, has been adopted for non-aureus species.^21,24^ In the case of methicillin-resistant *S*. *pseudintermedius* SCC*me*c type IV and V predominant.^12,25^

In addition to the challenge *S*. *pseudintermedius* and methicillin-resistant *S*. *pseudintermedius* presents in veterinary medicine, it is increasingly being recognized as a zoonotic human pathogen responsible for a range of infections, most commonly skin and soft tissue infections.^26^

Here we highlight the diversity of SCC*mec* elements encoded within staphylococci with the description of two novel SCC*mec* elements in *S*. *pseudintermedius*.

## Material and methods

### Ethics

Samples were collected through routine diagnostic procedures with the written informed consent of the owner and approved by the institutional Veterinary Ethical Review Committee (reference 28.21).

### Isolation, antimicrobial susceptibly testing and whole-genome sequencing

Canine *S*. *pseudintermedius* clinical isolates 6110-24416, 6127-64107, 7017-61515 and 10916-77753 were collected during routine veterinary diagnostic work at Easter Bush Pathology within the Royal (Dick) School of Veterinary Studies, University of Edinburgh, UK 7017-61515 came from a wound in 2016 and 10916-77753 from an infected digit in 2017. 6110-24416 and 6127-64107 we isolated from canine pyoderma in 2013 and 2016 respectfully. Identification and antimicrobial susceptibly testing was performed by Vitek^®^ 2 (bioMérieux, Basingstoke, UK) using the AST-GP80 card and applying Clinical and Laboratory Standards Institute (CLSI) Veterinary Interpretation Guidelines,^27^ antibiograms shown in Table S1 (available as Supplementary data at *JAC* Online), the oxacillin breakpoint being S ≤ 2 R ≥ 4 mg/L. Oxacillin disc diffusion was also performed using CLSI guidelines, the zone diameter breakpoint being S ≥ 18 R ≤ 17 mm.^27,28^ All four isolates were whole-genome-sequenced at MicrobesNG (Birmingham, UK).

### Genome assembly and SCC*mec* identification

Illumina HiSeq technology with 2 × 250 bp paired-end reads and read trimming was performed by MicrobesNG. To generate a fully assembled SCC*mec* additional long read sequencing with Oxford Nanopore technology was carried out on isolates 6127-64107 and 10916-77753, also performed by MicrobesNG. In either case, read trimming was achieved using Trimmomatic v.0.30^29^ using a sliding window quality cut-off of 15. *De novo* genome assembly was done using Unicycler v.0.4.8^30^ using default parameters. All assemblies were annotated using the NCBI Prokaryotic Genome Annotation Pipeline.^31^

Contiguous sequences (contigs) containing the *orfX* and *mecA* genes were identified using BLAST, using the respective genes from *S. aureus* N315 Type II SCC*mec* (accession number D86934) as query sequences. For 6110-2416 and 7017-61515, contig JASSUT010000010 and JASSUS010000005 were identified, respectively, as containing the *orfX* and *mecA* genes. Initial analysis of 6127-64107 and 10916-77753 identified *orfX* and *mecA* located on different contigs. To resolve the SCC*mec* sequence, subsequent long read sequencing was carried out. This resulted in complete genome sequences with *orfX* and *mecA* located at 32 319..32 795 and 64 798..62 789 bp, respectively, within the genome of 6127-64107 and for 10916-77753, *orfX* and *mecA*, were located at 3 316..32792 and 64 370..62 361 bp, respectively.

### Multi-locus sequence typing

Multi-locus sequence types (ST) were derived from the assembled genomes using the MLST tool available from the Center for Genomic Epidemiology (www.genomicepidemiology.org).^32^

### Nucleotide accession numbers

All genome sequences generated in this study have been deposited in GenBank under Bioproject PRJNA978491. Accession numbers for individual isolates are as follows: 6110-24416—Biosample: SAMN35555367, SRA: SRR24792123, Assembly: JASSUT000000000; 6127-64107—Biosample: SAMN35555368, SRA: SRR24792119 and SRR24792122, Assembly: CP127100; 7017-61515—Biosample: SAMN35555369, SRA: SRR24792121, Assembly: JASSUS000000000; 10916-77753—Biosample: SAMN35555370, SRA: SRR24792118 and SRR24792120, Assembly: CP127101. The assembled SCC*mec* regions of 6110-24416, 6127-64107, 7017-61515 and 10916-77753 generated in this work have been deposited under accession numbers, OR082610, OR082612, OR082611 and OR082613.

## Results and discussion

### The SCC*mec* of S. pseudintermedius 7017-61515 contains a novel *mec*complex and *ccr* type combination

*S. pseudintermedius* 7017-61515 was isolated from a canine wound with genome sequence-derived multi-locus sequence typing revealed this isolate to have a novel ST type, assigned ST1200. BLASTn analysis, with *mecA* from *S. aureus* N315, SCC*mec* Type II (accession D86934) as the query sequence, revealed carriage of *mecA* within the genome of 7017-61515 on JASSUS010000005. To identify whether isolate 7017-61515 carried any SCC*mec* elements, manual examination of the region surrounding the *mecA* gene of 7017-61515 was undertaken. This showed the presence of *mecR1* encoded upstream of *mecA* and on the opposite strand. Immediately downstream of *mecR1* was *mecI*. Downstream of *mecA* was a hypervariable region followed by the insertion sequence IS*431*, which contained a frame-shift mutation. These features, taken together, indicate that the *mecA* gene of 7017-61515 is encoded within a Class A *mec* gene complex.^33^ Indeed, the *mecA* complex of 7017-61515 shared 99.30% nucleotide identity with the Class A *mec* gene complex from the Type II SCC*mec* of *S. aureus* N315. The *mecA* region was located 951 bp downstream of *orfX*, a 23S rRNA methyltransferase gene, indicating it was encoded within a SCC*mec* element.^33^

To characterize the SCC*mec* element, a search for the *ccr* genes was carried out. A single *ccr* gene was identified 6847 bp downstream from the *mecA* complex and shares 98% nucleotide identity with *ccrC1* from the Type VII SCC*mec* of *S. aureus* PM1 (accession number AB462393). This constitutes a type 5 *ccr* gene complex.^33^ BLASTn analysis using the Class A *mec* gene complex as query, against the non-redundant nucleotide database, identified 500 staphylococci strains with this *mec* class and out of those only 118 had *ccrC1* associated. Of the typed SCC*mec* elements, Type III (accession number AB037671) and Type XIV (accession number LC440647.1) are the only elements that contain both a Class A *mec* gene complex and a type 5 *ccr* gene complex. However, both the Type III and Type XIV SCC*mec* elements have the type 5 *ccr* as part of a composite element with other *ccr* types; as yet, there has been no type classification of a SCC*mec* that contains both Class A *mec* gene complex with a type 5 *ccr*. To the best of our knowledge, this is the first description of this combination of *mec* class and *ccr* type from a non-composite element.

**Figure 1.**
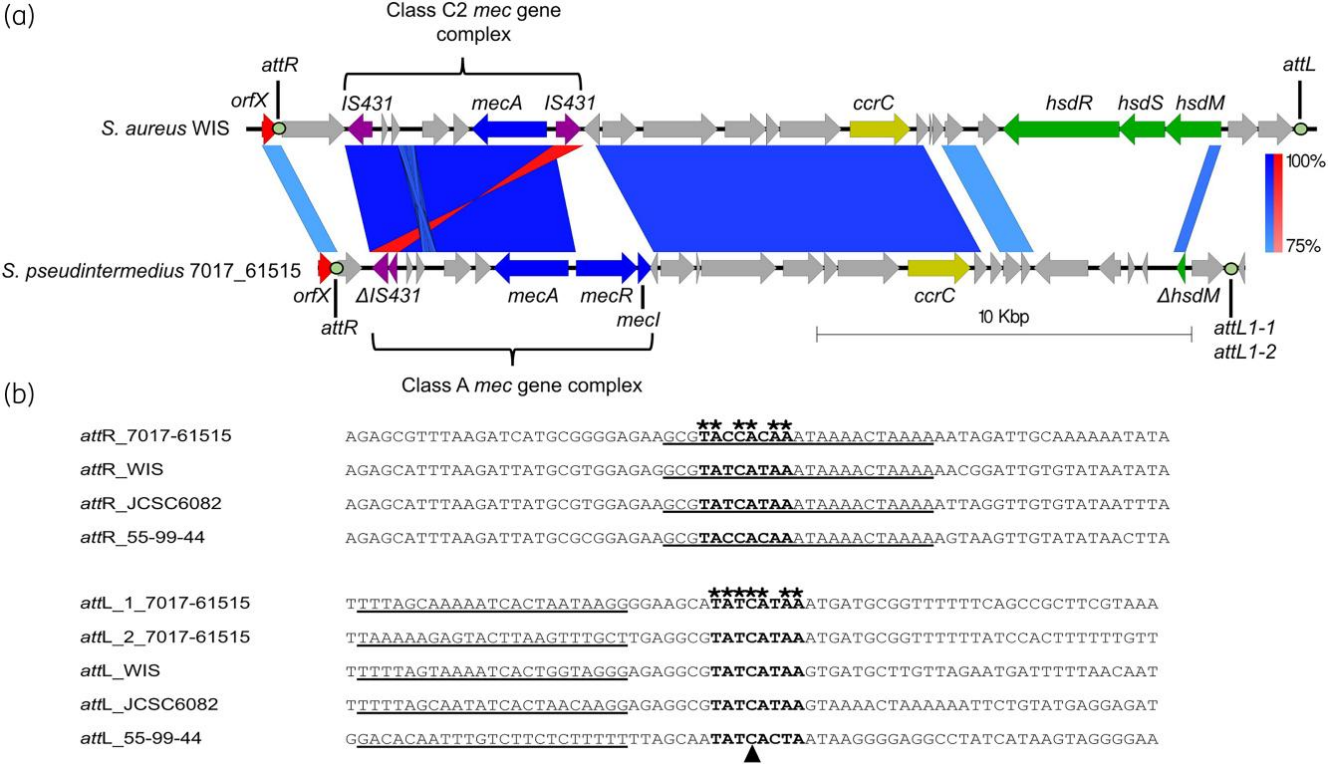
Overview of *S. pseudintermedius* 7017-61515 SCC*mec* and associated *att* sites. (a) *S. aureus* WIS corresponds to Type V SCC*mec*, accession number AB121219, with *S. pseudintermedius* 7017-61515 corresponding to this isolates SCC*mec* region. Bands connecting the two sequences represent regions of homology, with the percentage identity key shown on the right. Inverted sequence alignment is shown in red, normal sequence alignment is shown in blue. Key features associated with SCC*mec* elements are labelled. *att* sites are highlighted by filled circles and labelled above/below. (b) Comparison of *att*R/L sites of only *ccr*C-containing SCC*mec*. Conserved nucleotide bases are represented by an asterisk, for the core 8 bp region, which is represented in black, bold font. The central cytosine, indicated by a black triangle, thought to be essential for recombination between *att*B and *att*SCC.^34^ Inverted repeats are marked by the underlined bases. Sequences of known *att*R and *att*L sites associated with *ccr*C [from *S. aureus* WIS (WIS), *S. aureus* JCSC6082 (JCSC6082), AB373032; *S. aureus* 55-99-44 (55-99-44), MG674089] were aligned and compared with those identified in *S. pseudintermedius* 7017-61515. This figure appears in colour in the online version of *JAC* and in black and white in the print version of *JAC*.

Integration of SCC*mec* elements into the chromosome of staphylococci is achieved via an *attB* site, located at the 3′ end of the gene *orfX*.^34^ This integration results in the generation of direct repeat regions, *attR* and *attL*, which flank the element.^19^ Therefore, to establish the boundaries of the SCC*mec* element, the *orfX* region was searched to identify these repeat regions. The following search sequences, generated from previously identified *attR* and *attL* sequences, respectively, were used; gc[ag]tatca[tc]aaatgatgcggttt and aacc[tg]catca[tc][tc][at][ac]c[tc]gataag[ct]. The *attL* search sequence did not reveal any corresponding match, however, the *attR* search sequence gave two matches starting 23.7 kbp from the 3′-end of *orfX*. These two matches appear to be direct repeats reminiscent of known *attL* sites^35^ separated by 32 bp, and were assigned *attL1-1* and *attL1-2* [Figure 1(a and b)]. As the *attR* search sequence did not reveal an *attR* site within *orfX*, manual inspection of the 3′ end of the gene was carried out. From this inspection an *attR* sequence could be identified. This *attR* sequence differed from the *attR* sites used to generate the search sequence pattern, which were primarily associated with *ccrAB* carriage, with the *attR* of 7017-61515 being homologous to the *attR* sequences of SCC*mec* carrying only *ccrC* [Figure 1(b)]. Our previous work revealed that the *attR* sites differ between SCC*mec* that carry either *ccrAB* and *ccrC*, which would explain why the initial *attR* search sequence did not find a match within the 3′ end of the *orfX* gene.^35^ Further to this, the central 8 bp of the *attR* sequence of 7017-61515 did not contain the traditional TATCATAA conserved bases but TACCACAA. Although this is different from the previously identified central 8 bp sequences, the central cytosine, thought to be essential for *attB* and *att*SCC recombination, is still present.^34^ Therefore, the identified *attR* site from 7017-61515 was considered accurate, resulting in a SCC*mec* element of 24 779 bp.

SCC*mec*_7017-61 515_ contains three J regions, defined as the areas between the *orfX*, *mec* and *ccr* genes. The whole element shares 92% identity with the Type V SCC*mec* element from *S. aureus* WIS (accession number AB121219), although only 59% coverage [Figure 1(a)]. One region of similarity corresponds to the *mec* region, though this region varies between the two SCC*mec*, as the Type V SCC*mec* contains a Class C2 *mec* gene complex, rather than Class A. The other region of similarity is the J2 region containing *ccrC1*, as well as putative proteins. The J3 region shares the least amount of similarity, with the J3 region of 7017-61515 lacking the type 1 restriction-modification genes, with the exception of a truncated *hsdM* gene, present within the Type V SCC*mec* [Figure 1(a)].

Oxacillin disc diffusion following CLSI methodology, showed that all four study isolates were resistant. Interestingly, while 6110-24416, 6127-64107 were resistant to oxacillin (MIC ≤ 0.25 mg/L) using Vitek^®^ 2, 7017-61515 and 10916-77753 was susceptible to oxacillin, showing that phenotypic resistance could be overlooked by certain testing methodologies.

### Isolates 6110-24416, 6127-64107 and 10916-77753 contain a novel composite SCCmec

**Figure 2.**
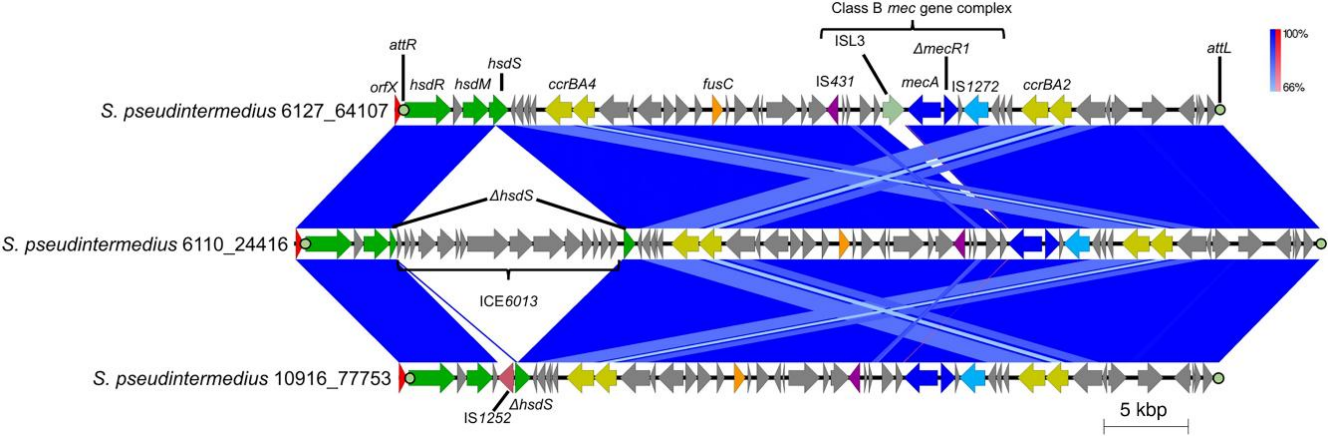
Overview of SCC*mec* elements in isolates 6110-24416, 6127-64107 and 10916-77753. The SCC*mec* region from each isolate is labelled with the corresponding name. Regions of homology are highlighted by the bands connecting the sequences, with the percentage identity key shown on the right. Inverted sequence alignment is shown in red, normal sequence alignment is shown in blue. Key features associated with SCC*mec* elements are labelled. *att* sites are highlighted by filled circles and labelled above/below. The *ICE*6013 of 6110-24416 has been labelled and lack of connecting bands indicates this region is missing from the SCC*mec* of 6127-64107 and 10916-77753. This figure appears in colour in the online version of *JAC* and in black and white in the print version of *JAC*.

*S. pseudintermedius* isolates 6110-24416, 6127-64107 and 10916-77753 were found to be methicillin resistant, with MLST revealing them to be ST561, ST41, ST668, respectively. Initial sequence analysis, as described before, revealed that all three isolates carried *mecA*. Manual inspection of the region surrounding *mecA* identified the presence of a truncated *mecR1*, with insertion sequence (IS) elements IS*431* and IS*1272*, upstream and downstream of *mecA*, respectively. Therefore, the *mec* complex encoded by all three isolates is Class B.^33^ All three isolates’ complexes shared 99% nucleotide identity with the Class B *mec* of *S. aureus* HDE288 (accession number AF411935), with 98% coverage for both 6110-24416 and 10916-77753, and 83% coverage for 6127-6107. The lower coverage for 6127-6107 was found to be due to the presence of an additional IS element, located immediately downstream of *mecA*, putatively associated with the ISL*3* family (Figure 2).

**Figure 3.**
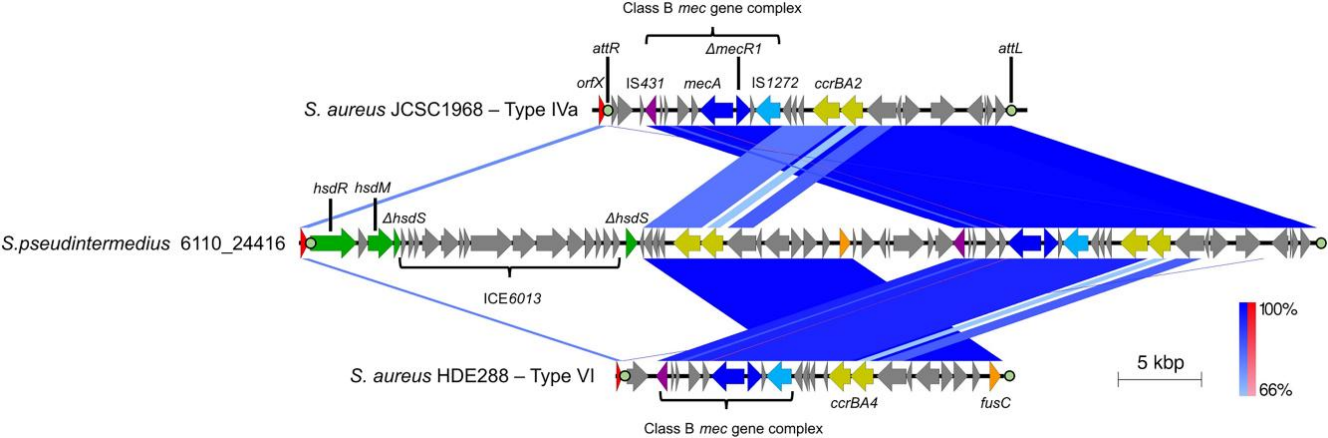
Similarity between the SCC*mec* region of 6110-24416 and Type IVa and Type VI SCC*mec*. The top sequence is the type IVa of *S. aureus* JCSC1968, accession number AB063172, followed by the SCC*mec* of *S. pseudintermedius* 6110-24416, with the bottom sequence corresponding to the Type VI *SCCmec* of *S. aureus* HDE288, accession number AF411935. Bands connecting to the middle sequence to the sequence above and below denotes regions of sequence homology. The percentage identity key is shown on the right, with normal sequence alignment represented by blue and inverted sequence alignment is shown in red. Key features associated with the SCC*mec* elements are labelled, including the *ICE*6013 region of 6110-24416. *att* sites are highlighted by filled circles and labelled above/below. This figure appears in colour in the online version of *JAC* and in black and white in the print version of *JAC*.

To determine which SCC*mec* type these isolates carried, identification of the *ccr* genes was undertaken. Two copies of both *ccrA* and *ccrB* were found, located 16 779 bp upstream and 1929 bp downstream of the *mec* complex (Figure 2). nBLAST analysis against previously identified *ccrAB* genes, revealed the *ccr* complex upstream was type 4, sharing 99% identity with *ccrAB4*, whereas the *ccr* complex downstream was type 2, sharing 97% identity with *ccrAB2*,^33^ suggesting the identified SCC*mec* may be a composite of Type IVa and VI (Figure 3). Supporting this hypothesis, is the presence of a *fusC* gene within the region containing the *ccrAB4* genes, which could be attributed to type VI SCC*mec* (Figure 3).

To establish the boundaries of the SCC*mec*, *att* sites were determined. Using the search sequence mentioned previously, an *attR* site was found within the 3′ end of the *orfX*, with an *attL* site discovered 60 391, 48 755 and 48 275 bp from *attR*, for isolates 6110-24416, 6127-6107 and 10916-77753, respectively (Figure 2). Resulting in SCC*mec* elements of 61 259 bp for isolate 6110-24416, with isolates 6127-6107 and 10916-77753 having elements of 49 294 bp and 48 809 bp in size, respectively.

The SCC*mec* within isolates 6110-24416, 6127-6107 and 10916-77753 share a significant amount of homology, however, due to the size discrepancies of the elements, further examination of the J regions was carried out. The J region between *orfX* and the *ccrBA4* genes contained type 1 restriction-modification genes, *hsdRMS*. However, only isolate 6127-6107 had an intact set of these genes, as isolate 10916-77753 had an IS element, IS*1252*, inserted into the *hsdS* gene, with isolate 6110-24416 having an integrative conjugative element (ICE) within its copy of *hsdS* (Figure 2). To further characterize the ICE of 6110-24416, comparative analyses were carried out with known ICE of other staphylococci. This analysis revealed that the ICE of 6110-24416 belongs to the *ICE6013* family (subfamily 3), as it shared 94% nucleotide coverage and 97% nucleotide identity with the ICE*6013* of *S. intermedius* NCTC 11048 (accession number UHDP00000000).^36^ Work carried out by Sansevere *et al.* demonstrated that ICE*6013*, of subfamily 1, can conjugatively transfer between different *S. aureus* backgrounds but did not detect any mobilization of chromosomal DNA.^36^ This suggests that the ICE*6013* within the SCC*mec* of 6110-24416 is unlikely to be involved in the mobilization of the SCC*mec*, it may still have an impact on the SCC*mec*’s ability to excise from the chromosome. However, it should be noted that ICE*6013* are diverse, with seven subfamilies described,^36^ allowing for the possibility that ICE*6013* from different subfamilies may mobilize chromosomal DNA on excision.

### Conclusion

Here, we present the discovery of two novel SCC*mec* elements within four clinical isolates of *S*. *pseudintermedius* from companion dogs. This finding expands our knowledge of the diverse range of this important staphylococcal antimicrobial resistance determinant, with relevance for both human and veterinary medicine. However, to gain a comprehensive understanding of atypical SCC*mec* elements, further research is warranted to elucidate their diversity, epidemiology and implications for understanding antimicrobial resistance effectively.

## Supplementary Material

dkae096_Supplementary_Data

## Supplementary data

Table S1 is available as Supplementary data at *JAC* Online.
