# Supplementary material for: Methicillin resistance in Staphylococcus pseudintermedius encoded within novel staphylococcal cassette chromosome mec (SCCmec) variants
Source: J Antimicrob Chemother. 2024 Apr 2;79(6):1303–8. doi: 10.1093/jac/dkae096 (PMC11144489; doi:10.1093/jac/dkae096)
Supplement: dkae096_Supplementary_Data [file dkae096_supplementary_data.docx]

Table S1 Antibiogram of Study Isolates

|  | Interpretive Categories and MIC Breakpoints, mg/L * | 6110-24416 | 6127-64107 | 7017-61515 | 10916-77753 |
| --- | --- | --- | --- | --- | --- |
| Penicillin G | S <0.125 R >0.25 | R | R | R | R |
| Oxacillin | S <0.25 R >0.5 | R | R | S | S |
| Gentamicin | S <4 R >16 | S | S | S | S |
| Enrofloxacin | S <0.5 R >4 | S | S | S | S |
| Marbofloxacin | S <1 R >4 | S | S | S | S |
| Pradofloxacin | S <0.25 R >2 | S | S | S | S |
| Erythromycin | S <0.5 R >8 | R | R | R | R |
| Clindamycin | S <0.5 R >4 | R | R | S^+^ | R |
| Inducible clindamycin resistance |  | negative | negative | positive | negative |
| Tetracycline | S <0.25 R >1 | S | S | S | S |
| Nitrofurantoin | S <32 R >128 | S | S | S | S |
| Chloramphenicol | S <8 R >32 | S | R | S | R |
| Trimethoprim- sulfamethoxazole | S <2/38 R >4/76 | S | S | R | S |
| Performed using performed by Vitek^®^2 (bioMérieux, Basingstoke, U.K.) using the AST-GP80 card. *Performance Standards for Antimicrobial Disk and Dilution Susceptibility Tests for Bacteria Isolated From Animals. 5th ed. CLSI supplement VET01S. Clinical and Laboratory Standards Institute; 2020. R = resistance, S = sensitive. ^+^ note positive for inducible clindamycin resistance | | | | | |
